# Concordance between the TIRADS ultrasound criteria and the BETHESDA cytology criteria on the nontoxic thyroid nodule

**DOI:** 10.1186/s13044-017-0037-2

**Published:** 2017-02-02

**Authors:** Hernando Vargas-Uricoechea, Ivonne Meza-Cabrera, Jorge Herrera-Chaparro

**Affiliations:** 1 Department of Internal Medicine, Division of Endocrinology and Metabolism, Universidad del Cauca, Popayán, Colombia; 2 Laboratory of Pathology, Hospital Universitario San José, Popayán, Cauca Colombia; 3 Department of surgery, Universidad del Cauca, Carrera 6 No 41 N-135 apto 202B terrazas del campestre, Popayán, Cauca Colombia

**Keywords:** Thyroid, Nodule, TIRADS, Bethesda, Concordance

## Abstract

**Background:**

Thyroid nodule is a common disorder of the thyroid. Despite their benign nature, they can be associated with multiple pathologic conditions, including thyroid cancer.

**Methods:**

This cross-sectional study determined the concordance of Ultrasound (TIRADS criteria) and Fine Needle Aspiration Biopsy (FNA-BETHESDA system) in the assessment of the nontoxic thyroid nodule. A total of 180 subjects 18 years old or older underwent the two diagnostic tests and their results were compared using kappa index.

**Results:**

Participants were mostly women, with average age of 57 years. The frequency of BETHESDA II was 65/180 versus 45/180 in TIRADS 2. In contrast, the highest frequency in category 4-IV was 62/180 for TIRADS 4 versus 41/180 for BETHESDA IV. The highest concordance was found among the category 2-II classification. The observed agreement was 87.2% with a linear weighted kappa of 0.69 (95% CI: 0.59-0.79). The heterogeneity analysis showed a trend towards a higher weighted kappa value in nodules ≥4 cm in males and individuals aged ≥50 years, with accelerated nodular growth, binding to adjacent structures, vocal folds paralysis, urban origin, and a history of head and neck radiation therapy.

**Conclusions:**

The TIRADS criteria has a good concordance with the Bethesda system. The ultrasound findings of benign pathology are aligned with the cytology results. The correct interpretation of the two findings helps the clinician to reduce the risk of unnecessary invasive procedures in patients with a low probability of presenting thyroid cancer, while facilitating the identification of patients at higher risk of cancer.

## Background

A steady increase in the incidence rate of thyroid cancer has been noted in recent decades all over the world, and the causes of this increase are still controversial. Thyroid cancer is the most common endocrine malignancy (1.0–1.5% of all newly diagnosed cancers in the United States of America every year are originally thyroid). The increased frequency in thyroid cancer is almost exclusively due to the rise in the number of papillary cancers, with no significant changes in other histologic subtypes [1, 2]. The typical presentation is as small tumors, though there is a growing incidence of large tumors; it has been hypothesized that the rise in the incidence of thyroid cancer is mostly due to improved detection rather than to a real increase in frequency [3]. Thyroid nodule can be defined as a discrete lesion within the thyroid gland that is radiologically distinct from the surrounding thyroid parenchyma. It may be solitary, multiple, solid, or cystic, and may or not be functional. Thyroid nodules are frequent among the general population and thyroid Ultrasound (US) has considerably increased the number of cases identified. Thyroid nodules may be palpated in about 4–8% of the general population (however, neck palpation is very imprecise in terms of determining the size and morphology). US identifies the presence of nodules in 19-67% of the cases, and is an accurate method for the detection of thyroid nodules; however, US has a low accuracy in differentiating between benign from malignant thyroid nodules [4]. The sonographic characteristics of a thyroid nodule associated with a higher likelihood of malignancy include hypoechogenicity, increased intranodular vascularity, irregular margins, microcalcifications, absent halo, and a taller-than-wide shape measured in the transverse dimension. Thus, several benign and malignant ultrasound gray scale and Doppler features have emerged over the last ten years that may be used in different ways to assign probabilities, together with a method based on the Breast Imaging Reporting and Data System (BIRADS). Likewise, several US Thyroid Imaging Reporting and Data Systems (TIRADS) have been proposed for risk stratification of thyroid nodules [5].

The nodules are usually divided into different categories based on TIRADS and are then referred for Fine-Needle Aspiration (FNA) Biopsy or follow-up, according to the variable risk of malignancy. The terminology of TIRADS was first used by Horvath et al. [6]. They described 10 US patterns of thyroid nodules and related the rate of malignancy based on the pattern. The initial purpose of TIRADS was to improve patient management and cost-effectiveness by avoiding unnecessary FNA Biopsies in patients with thyroid nodules (Table 1), with a sensitivity, specificity, positive predictive value, negative predictive value, and accuracy of 88, 49, 49, 88, and 94%, respectively. However, its clinical use is still very limited and its practical application in clinical practice is questioned. Moreover, FNA Biopsy is the most accurate method for determining malignancy, and is a fundamental part of current thyroid nodule evaluation. The Bethesda System for Reporting Thyroid Cytopathology is a standardized reporting system for classifying thyroid FNA Biopsy results that comprises six diagnostic categories with unique risks of malignancy and recommendations for clinical management. Since its inception, the Bethesda System has been widely adopted, each category conveys a risk of malignancy and recommended next steps, though it is unclear if each category also predicts the type and extent of malignancy (Table 1). Nevertheless, the implementation of this reporting system has shown significant diagnostic variability, both inter and intra pathologists, particularly when read as “atypical cells of undetermined significance, follicular lesion of undetermined significance, or follicular neoplasm” (also termed as Bethesda Category III, comprising a heterogeneous population of low-risk lesions that contain follicular cells exhibiting either architectural abnormalities or nuclear atypia that do not fit into other definitive cytological categories). A recent meta-analysis evaluated the validity of the Bethesda reporting system and found 97% sensitivity, 50.7% specificity and 68.8% diagnostic accuracy; the negative and positive predictive values were 96.3 and 55.9%, respectively [7, 8]. Notwithstanding the fact that both US and FNA biopsy are widely recommended procedures to study patients with thyroid nodules, the value of the existing concordance between the two methods has not been established. Consequently, the purpose of this study was to assess the existing concordance between the two diagnostic methods used in the initial evaluation of individuals with non-toxic thyroid nodule (TIRADS and Bethesda systems).

**Table 1:** Thyroid imaging reporting and data system (TIRADS) and the Bethesda System for Reporting Cytopathology (ref. 6, 8, 9)

TIRADS		BETHESDA	
Categories	Features	Diagnostic Categories	Risk of malignancy
TIRADS 1	Normal thyroid gland.	I. Nondiagnostic or unsatisfactory.	-
TIRADS 2	Benign conditions (0% malignancy).	Cyst fluid only.	
TIRADS 3	Probably benign nodules (5% malignancy).	Virtually acellular specimen.	
TIRADS 4	Suspicious nodules (5–80% malignancy rate). A subdivision into 4a (malignancy between 5 and 10%) and 4b (malignancy between 10 and 80%) was optional.	Other (obscuring blood, clotting artifact, etc.).	
TIRADS 5	Probably malignant nodules (malignancy >80%).	II. Benign.	0-3
TIRADS 6	Category included biopsy proven malignant nodules.	Consistent with a benign follicular nodule (includes adenomatoid nodule, colloid nodule, etc.).	
		Consistent with lymphocytic (Hashimoto) thyroiditis in the proper clinical context.	
		Consistent with granulomatous (subacute) thyroiditis.	
		III. Atypia of undetermined significance/follicular lesion of undetermined significance.	5-15
		IV. Follicular neoplasm/"suspicious" for follicular neoplasm. Specify if Hürthle cell type.	15-30
		V. Suspicious for malignancy.	60-75
		Suspicious for papillary carcinoma.	
		Suspicious for medullary carcinoma.	
		Suspicious for metastatic carcinoma.	
		Suspicious for lymphoma.	
		VI. Malignant.	97-99
		Papillary thyroid carcinoma.	
		Poorly differentiated carcinoma.	
		Medullary thyroid carcinoma.	
		Undifferentiated (anaplastic) carcinoma.	
		Squamous cell carcinoma.	
		Carcinoma with mixed features.	
		Metastatic.	

## Methods

The overall objective of the study was to determine the level of concordance between the ultrasound criteria established under TIRADS (The Thyroid Imaging Reporting and Data System for US of the thyroid); and the cytology criteria according to The Bethesda System for Reporting Thyroid Cytopathology [9, 10]. Additionally, the study population was characterized from the socio-demographic point of view, the concordance of the classification systems was estimated, and the heterogeneity of the factors influencing the consistency of the various classification systems was analyzed.

### Ethics approval and consent to participate

All personal data were confidential and managed exclusively by the principal investigator, according to the legal standards on the confidentiality of the medical record and adhering to the rules of the Institutional Review Committee of Human Ethics (reference number: 221–011). Universidad del Valle, Valle del Cauca-Colombia.

#### Design of the study

This was a cross-sectional study to evaluate the concordance between two diagnostic systems (TIRADS and Bethesda), administered simultaneously to the same individual. The population consisted of consecutive patients consulting the outpatient endocrinology, internal medicine, or general surgery departments at a high complexity referral center, with a diagnosis of nodular or non-nodular “thyroid dysfunction”. The inclusion criteria were as follows: male and females aged 18 years and older, with a non-toxic thyroid nodule (ranges for normal thyroid tests were Thyrotrophin (TSH): 0.4 to 4 mIU/L; Free thyroxine: 0.8 to 1.8 ng/dL, according to the National Academy of Clinical Biochemistry) identified either clinically or through imaging [11]. The exclusion criteria were: TIRADS 1 and Bethesda I (Table 1); Graves-Basedow–associated hyperthyroidism, patients with toxic thyroid nodular disease, chronic hypothyroidism (with a minimum of six-months on treatment with levothyroxine sodium), iatrogenic hyperthyroidism resulting from high-dose sodium levothyroxine therapy regardless of the indication; a history of surgically resected thyroid cancer, and patients with a history of partial thyroidectomy (lobectomy) or subtotal/near total thyroidectomy under levothyroxine sodium therapy (the latter criterion is based on the fact that a constant high stimulus of thyroid hormones and the concomitant TSH suppression in patients with endogenous hyperthyroidism and levothyroxine management may impact the size of the thyroid nodules) [12, 13].

This study was supported by the Internal Medicine Department from The Faculty of medicina of the Universidad del Cauca (Popayán-Colombia), who provided funding to conduct the analysis and prepare the manuscript.

#### Sample size estimate and sampling

To estimate the sample size, matched categories in both reporting systems were considered. Based on the data from a pilot study with 32 subjects that met the above selection criteria, and using the formula below, the N value was established at 128 subjects:

$$ n=\frac{P_e}{e{e}^2\left(1-{P}_e\right)} $$

Where:

P_e_: Expected percentage of random concordance

*Ee*: Kappa index standard error [14–16].

A consecutive non-probabilistic sampling was used based on an initial review of 217 medical records; however the final analysis was limited to 180 patients and 37 patients were excluded due to:

1. Incomplete family history and missing socio-demographic information in 26 records.
2. The echography was not reported according to TIRADS criteria in 4 records.
3. The cytology results were not reported according to the Bethesda criteria in 5 cases.
4. The ultrasound examination had been done at a different institution or by a different radiologist in 2 cases.

The source of the information in this study is a registry of consecutive data from an outpatient center for patients with a diagnosis of thyroid dysfunction. A standard form collected socio-demographic information, family and personal history of diseases, in addition to the data available from the medical record. Patients undergoing thyroid ultrasound imaging and FNA Biopsy due to non-toxic nodular thyroid disease were analyzed in accordance with the medical opinion of the institution’s study group on thyroid disease (endocrinology, pathology, radiology and surgery). All patients were informed about the procedure and after signing the informed consent, the thyroid ultrasound was performed, and the node(s) were sampled according to Crockett’s FNA Biopsy protocol [17].

The same radiologist read all the tests. One out of every 20 patients was randomly selected to repeat the ultrasound examination. The principal researcher interpreted the results in accordance with the TIRADS criteria and if the second reading was inconsistent with the first, a second radiologist was asked for an opinion to arrive at a consensus between the two radiologists and establish a TIRADS-based ultrasonographic diagnosis. 9 of the 180 participants were randomly selected to assess the radiologists’ agreement. One of the nine US results showed disagreement because the first radiologist reported TIRADS 3, while the second one reported TIRADS 2, based on the original classification. Upon further analysis the conclusion was TIRADS 3. The material obtained via the FNA Biopsy was placed on a glass slide previously impregnated with 96% alcohol and then a second glass slide was placed on top. The smear was again immersed in 96% alcohol and then stained using the Papanicolaou technique. To ensure the quality of the cytology specimens, the same experienced pathologist read the slides and reported a diagnosis based on the Bethesda criteria. One out every ten specimens was randomly selected to be analyzed by a second pathologist. In case of disagreement between the two pathologists, a pathologist meeting was convened (five pathologist). The second pathologist disagreed with two of the 18 specimens subject to a second evaluation; in both cases, the first pathologist classified the cytology specimen as Bethesda V, while the second pathologist classified the specimens as Bethesda VI. Both specimens were further evaluated at a pathologist meeting, and the final classification was Bethesda VI. The radiologists and the pathologists were blinded to the patients’ medical record data for both the ultrasound examination and the FNA biopsy.

#### Statistical analysis

The weighted Kappa statistical method with a 95% confidence interval and the statistical Z-test were used to estimate the level of concordance between the two systems. In order to pursue the Kappa analysis, categories 5 and 6 of both the TIRADS and the Bethesda classification were combined since the highest risk for malignancy is usually described in these two categories. Category 1 in both classifications was excluded from the selection process because a TIRADS 1 ultrasound examination is considered normal, and Bethesda I is considered an unsatisfactory specimen. The purpose of excluding category 1 was to avoid invalidating further comparisons since category 1 is inconclusive, particularly Bethesda I. Consequently, the analysis categories are as follows:

- TIRADS 2: “BENIGN”
- TIRADS 3: “PROBABLY BENIGN”
- TIRADS 4: “SUSPICIOUS”
- TIRADS 5: “PROBABLY MALIGNANT”
- Bethesda II: “BENIGN”.
- Bethesda III: “PROBABLY BENIGN”.
- Bethesda IV: “SUSPICIOUS”.
- Bethesda V: “PROBABLY MALIGNANT”

Weighted Kappa statistic with linear weight was used to estimate the level of agreement between the two systems; Kappa with quadratic weighting was used for comparative purposes. A descriptive analysis was used to indicate the distribution of the quantitative variables. Based on that distribution, the average represented the central trend and the scatter represented the standard deviation. The qualitative variables were defined in terms of percentages by category. A stratified analysis was performed to explore heterogeneity factors, resulting in a linear weighted Kappa for the following categories: Gender, age, nodule size, urban/non-urban origin, accelerated nodule growth, vocal folds paralysis, hard nodule, attached to underlying structures, history of head and neck radiation therapy, and family history of thyroid cancer. All the analyses used STATA 10.1

## Results

The average age was 57 years old. Over 75% of the participants were females and 68.9% came from the urban area; however, there was a remarkable high frequency of risk factors for thyroid cancer. (Table 2) The frequency distribution according to the scales was strikingly different for categories 2-II and 4-IV. The frequency of category II in Bethesda was 65/180 versus 45/180 in TIRADS 2. In contrast, the highest frequency in category 4-IV was 62/180 for TIRADS 4 versus 41/180 for Bethesda IV. (Table 3) The highest concordance was found for categories TIRADS 2-Bethesda II (23.33%). None of the patients classified as TIRADS 2 were rated as Bethesda IV or V. In contrast, 4 subjects classified as Bethesda II were classified as TIRADS 4 (*n* = 2) or V (*n* = 2). Of the 35 patients classified as Bethesda V none were classified as TIRADS 2 or 3, but 3 of the 32 subjects with TIRADS 5 were classified as Bethesda II (*n* = 2) or III (*n* = 1). The weighted Kappa value according to the linear weights was 0.69 (95% CI: 0.59–0.79). The overall Kappa and the Kappa with quadratic weighting were also estimated for comparative purposes. (Table 4) The heterogeneity analysis showed a trend towards a higher weighted kappa value in nodules ≥4 cm in males and individuals aged ≥50 years, with accelerated nodular growth, binding to adjacent structures, vocal folds paralysis, urban origin, and a history of head and neck radiation therapy (Tables 5 and 6).

**Table 2:** Socio-demographic characteristics and risk factors for thyroid cancer

Characteristic	Frequency
Mean age	57 y (SD:±14y)
Sex	Fem: 141 (78.3%)
	Male: 39 (21.7%)
Origin	Urban: 124 (68.9%).
	Non-Urban: 56 (31.1%).
Thyroid cancer family backgrounds	N (%)
No	119 (66.1%)
Yes	61 (33.9%)
Accelerated Growth of the Thyroid Nodules	
No	121 (67.2%)
Yes	59 (32.8%)
Head Radiation	
No	163 (90.6%)
Yes	17 (9.4%)
Firm Nodule	
No	94 (51.7%)
Yes	86 (47.8%)
Adjacent structure Attachment	
No	130 (72.2%)
Yes	50 (27.8%)
Vocal Chord Paralysis	
No	130 (72.2%)
Yes	50 (27.8%)
≥4 cm nodule	
No	109 (60.6%)
Yes	71 (39.4%)

**Table 3:** Joint distribution of BETHESDA & TIRADS categories

Diagnostic categories		TIRADS				Total
		2	3	4	5	
BETHESDA	II	42	19	2	2	65
		23.33%	10.56%	1.11%	1.11%	
	III	3	21	14	1	39
		1.67%	11.67%	7.78%	0.56%	
	IV	0	1	33	7	41
		0%	0.56%	18.33%	3.89%	
	V	0	0	13	22	35
		0%	0%	7,22%	12,22%	
	Total	45	41	62	32	180

**Table 4:** Kappa comparison according to the estimation method

Kappa	Observed Agreement	Expected agreement	Kappa	Standard error	IC 95%	Z	Value p
Global	65.56%	25.27%	0.5391	0.0425	0.46–0.62	12.68	<0.001
Weighted (linear weights)	87.22%	58.76%	0.6901	0.0528	0.59–0.79	13.07	<0.001
Estimated (quadratic weights	94.63%	72.86%	0.8021	0.0731	0.66–0.94	10.97	<0.001

**Table 5:** Stratification according to nodule size, sex, and age in order to assess heterogeneity

Strata	Observed Agreement	Expected Agreement	Kappa	Standard Error	IC 95%
Nodule Size					
<4 cm	85.63%	65.15%	0.5876	0.067	0.46-0.72
≥4 cm	89.67%	66.26%	0.6938	0.083	0.53-0.86
Sex					
Men	89.74%	58.32%	0.7539	0.117	0.53-0.98
Women	86.52%	59.30%	0.6689	0.059	0.55-0.78
Age					
<50 years old	82.49%	59.91%	0.5632	0.092	0.38-0.74
≥50 years old	89.53%	59.18%	0.7435	0.064	0.62-0.87

**Table 6:** Heterogeneity assessment by stratifying the variables according to: thyroid cancer family history, accelerated growth of the nodules, firm nodule, underlying structure, vocal chords paralysis, origins, and history of radiation

Strata	Observed Agreement	Expected Agreement	Kappa	Standard Error	95% CI
Thyroid Cancer Family history					
Yes	87.98%	60.71%	0.694	0.086	0.53**–**0.86
No	86.83%	58.81%	0.680	0.066	0.55**–**0.81
Accelerated Growth of the nodule					
Yes	90.40%	60.12%	0.759	0.090	0.58**–**0.94
No	85.67%	59.89%	0.643	0.065	0.52**–**0.77
Firm Nodule					
Yes	86.82%	58.55%	0.682	0.076	0.53**–**0.83
No	87.59%	60.10%	0.689	0.073	0.55**–**0.83
Adjacent Structure Attachment					
Yes	94.00%	65.52%	0.826	0.096	0.64**–**1.01
No	84.62%	59.42%	0.621	0.062	0.50**–**0.74
Vocal Chords Paralysis					
Yes	94.67%	66.11%	0.843	0.096	0.65**–**1.03
No	84.36%	58.52%	0.623	0.062	0.50**–**0.74
Origin					
Urban (exclusive)	88.70%	58.99%	0.7245	0.065	0.60**–**0.85
Urban or Rural	84.41%	58.26%	0.6265	0.0881	0.45**–**0.8
Head and neck radiation therapy					
Yes	96.08%	61.48%	0.8982	0.1689	0.57**–**1.23
No	86.30%	58.55%	0.6695	0.0557	0.56**–**0.78

## Discussion

This study evaluated the concordance between the TIRADS and the Bethesda reporting systems on the non-toxic thyroid nodule. The result showed a “good or substantial” concordance and the most frequent consistency was found for categories II and IV. The kappa index measures the level of inter-observer concordance, or as in this particular case, the concordance between two diagnostic methods rather than the “quality” of the observation, so it is not possible to establish the validity of the resulting classifications. This study addresses the level of discrepancy, the report categories, and which categories tend to exhibit a higher frequency of discrepancies between the two methods. When particular types of disagreements are more frequent, this information shall be kept in mind when developing the kappa index [18, 19]. For this reason, the weighted kappa analysis was used, without neglecting the fact that although using weights is logical and attractive, it introduces a component of subjectivity since assigning weights is subjective and may impact the interpretation of the data when used for a different population –the weights assigned may vary based on the frequency of the disease-. This is evidenced through the variation in the kappa estimates when weighing is used, and depends on the weighing method used. The weighted kappa estimate with linear weights assigned to the categories shows a value of 0.69. The weighted kappa value based on quadratic weights was higher than the overall kappa or the linear weighted kappa (the quadratic weighted kappa value was 0.80). The difference is based on the fact that the linear and quadratic methods are based on the relative separation among the classification categories but the quadratic approach uses square differences, while the linear approach uses absolute values [20, 21]. Consequently, quadratic weights tend to assign a higher weight to disagreements that were relatively few in this study; when the kappa interpretation is based on quadratic weights, the level of concordance remains unchanged versus the interpretation of the linear weighted kappa; but if analyzed as an absolute value, it is evidently overestimated. Since the kappa value is affected by the prevalence of the characteristic studied, caution is of the essence when generalizing the results of inter-observer comparisons in the presence of varying prevalence. The prevalence of malignancy based on cytology findings (Bethesda V in the matched scale) was reported at 19.4% (35/180); however, using the TIRADS scale (maximum value of 5 in the matched scale), the prevalence of malignancy was 17% (32/180), showing a non-significant difference between the two methods. This is extremely relevant when considering that a prevalence of close to 50% results in a higher kappa value for the same proportion of agreements observed [22, 23]. Thus, the interpretation of the kappa index requires identifying the value of the marginal frequencies on the table (prevalence observed per observer). Since the difference between the prevalence estimated by both methods is not significant, the conclusion is than that the prevalence of the event did not affect the kappa value reported. When evaluating heterogeneity based on characteristics such as gender, age, size of the nodule, place of origin, accelerated nodular growth, vocal folds paralysis, hard nodule, binding to adjacent structures, a history of head and neck radiation therapy, a family history of thyroid cancer, the trend indicates a stronger concordance (expressed as a weighted kappa value). This is also the case for variables such as nodule size ≥4 cm, male gender, and age ≥50 years. Despite this trend, the study failed to show statistically significant differences. The TIRADS classification attempts to improve the interpretation of the findings of a thyroid nodule by defining categories that in the end are exclusive, although the original classification indicates a risk of malignancy between 5-80% for TIRADS 4, and this fact makes it difficult to clinically define a follow-up and management strategy. Notwithstanding this consideration, from the clinical perspective, in a subject with low probability of having thyroid cancer (and a TIRADS 2 or 3) the US negative predictive value will be greatly enhanced. The best US diagnostic performance is probably with extreme results of the classification (TIRADS 2–3 and TIRADS 5–6 of the original classification). Depending on the clinical probability of malignancy, the US findings may be more or less useful and applicable [24].

Previous studies have evaluated the diagnostic performance of both US and FNA Biopsy in the initial study of thyroid nodules. A recent study was aimed at developing a diagnostic algorithm using the data reported in the US (in accordance with a scoring system evaluating the risk of malignancy based on several US patterns) and the results of the FNA Biopsy (according to Bethesda). This study showed that classifying an individual in accordance with the presence of different US patterns as low, intermediate or high risk, together with the results of the FNA Biopsy, enables optimal clinical decision-making with regards to treatment strategies [25]. Along the same lines, other studies classify the risk of malignancy in accordance with the US characteristics and based on such risk, establish the need to perform a FNA Biopsy. The higher the risk of malignancy (according to the US) the greater the need to do the FNA Biopsy, and vice-versa –the lower the risk of malignancy based on the US, the lower the indication for a FNA Biopsy– [26–28].

Our study showed that the highest concordance was found among both the lowest risk (TIRADS 2 and Bethesda II) and the higher risk categories (TIRADS 4 and Bethesda IV), which is consistent with the previously described trials. This indicates that the US characteristics suggesting a higher or lower risk of malignancy, will be associated with higher or lower probability of malignancy according to the FNA Biopsy report (Bethesda), respectively.

Finally, the interpretation of the results in this study requires acknowledging that over two thirds of the subjects were women. Probably this trend is due to the fact that autoimmune thyroid disease is significantly more frequent in females than in males, so these patients with autoimmune thyroid disease visit the physician more often increasing the probability of detecting the nodules either through palpation or ultrasound; clinically this situation may be defined as a “medical surveillance bias” [29, 30]. The geographical distribution indicates that most of the patients were from urban areas and those from the rural areas were mostly from municipalities with accessible specialized care. The participants in the study had information about exposure/disease since they had been referred for a study of the thyroid nodule with a probable diagnosis of malignancy. In cross-section studies the participants may be more prone to participate based on their knowledge about exposure and disease and the convenience of their geographical location leading to a higher “selection bias” that in turn could overestimate the frequency of malignancies [31, 32]. This study highlights the high frequency of factors that have been historically associated with thyroid cancer. Those factors were evaluated with the survey administered to the study subjects that had been previously referred for tests to rule out malignancies, so these participants were more likely to recall past exposures (accurate or vague) potentially leading to a “recall bias” [30, 33]. Furthermore, since the data collection from the participants was not masked (they had been previously identified as nodular thyroid disease patients screened for malignancies), the interviewer’s interest in evaluating the exposure factors could have resulted in an “interviewer bias” [34, 35].

## Conclusions

The thyroid ultrasound report using the TIRADS criteria has a good concordance with the Bethesda cytology findings using FNA Biopsy. The ultrasound findings of benign pathology are aligned with the cytology results and vice-versa; ultrasound findings of malignancy shall be consistent with cytology-identified malignant disease. The correct interpretation of the two findings helps the clinician to reduce the risk of unnecessary invasive procedures in patients with a low probability of presenting thyroid cancer, while facilitating the identification of patients at higher risk of cancer. There is a need to develop study and monitoring protocols for cases classified as “discordant”, particularly when extreme categories are identified (TIRADS 5-Bethesda II, TIRADS 2-Bethesda V).

## Abbreviations

BIRADS: Breast Imaging Reporting and Data System
FNA: Fine-Needle Aspiration
TIRADS: The Thyroid Imaging Reporting and Data System for US of the thyroid
TSH: Thyrotrophin
US: Ultrasound
